# Supplementary material for: Metabolic Adaptation to Sulfur of Hyperthermophilic Palaeococcus pacificus DY20341T from Deep-Sea Hydrothermal Sediments
Source: Int J Mol Sci. 2020 Jan 6;21(1):368. doi: 10.3390/ijms21010368 (PMC6981617; doi:10.3390/ijms21010368)
Supplement: Supplementary file 1 [file ijms-21-00368-s001.pdf]

Supplemental Materials

Table S1. General features of the *Pa.pacificus* genome with other Thermococcales strains.

Parameters	Strains		
	Pa. pacificus	***P. furiosus***	***T. onnurineus***
**Genome size (bp)**	1,859,370	1,908,256	1,847,607
**Protein-coding regions (%)**	78.11	92.5	90.1
**GC content (%)**	43.04	40.8	51.0
**CDSs (no.)**	2,001	2,065	1,976
**tRNAs (no.)**	46	46	46
**rRNAs**	5S (2), 7S, 16S, 32S	5S (2), 7S, 16S, 32S	5S (2), 7S, 16S, 32S

Table S2. *Pa.pacificus* genes discussed in the text.

TRMS, strain DY20341 grown with the elemental sulfur; TRM, strain DY20341 grown without sulfur. RPKM, reads per kb per million reads. The log2 ratio of TRMS-RPKM/TRM-RPKM.

**Metabolic process**		**Gene ID**	**TRMS-RPKM**	**TRM-RPKM**	**log2(TRMS/TRM)**	**Predicted protein**
Central metabolism	Pentose-phosphate metabolism	PAP_06885	2706	2923	-0.11	Ribulose bisphosphate carboxylase(typeIII)
		PAP_04670	1601	1118	0.52	AMP phosphorylase (DeoA)
		PAP_04815	379	481	0.34	Ribose-1,5-bisphosphate isomerase (RBPI)
	Embden-Meyerhof pathway	PAP_01430	706	1271	-0.85	ADP-dependent glucokinase (GLK)
		PAP_01425	705	1174	-0.74	Phosphoglucose isomerase (PGI)
		PAP_04875	724	732	-0.02	ADP-dependent phosphofructokinase (PFK)
		PAP_03775	495	676	-0.45	Fructose-1,6-bisphosphate aldolase (FBA)
		PAP_09970	1092	950	0.20	Triosephosphate isomerase (TIM)
		PAP_09040	3732	1761	1.08	Glyceraldehyde-3-phosphate:ferredoxin oxidoreductase (GAPOR)
		PAP_03390	67	356	-2.41	Non-phosphorylating glyceraldehyde-3-phosphate dehydrogenase (GAPN)
		PAP_02855	1803	1268	0.51	NAD(P)-dependent glyceraldehyde 3-phosphate dehydrogenase(GAPDH)
		PAP_01540	781	768	0.02	2-phosphoglycerate kinase(PGK)
		PAP_01310	1902	1236	0.62	Phosphoglycerate mutase (PGM)
		PAP_04825	1226	945	0.38	Phosphoglycerate mutase (PGM)
		PAP_04410	4896	3069	0.67	Enolase (ENO), phosphopyruvate hydratase
		PAP_08515	1342	1075	0.32	Pyruvate kinase (PYK)
		PAP_01340	411	712	-0.79	Phosphoenolpyruvate synthase (PPS)
Metabolism of proteins	Utilization of proteins	PAP_05645 - 05675	327	334	-0.03	2-oxoglutarate oxidoreductase(OGOR)
			1074	1156	-0.11	
			624	684	-0.13	
			373	295	0.34	

		1042<br>605<br>466	1109<br>641<br>771	-0.09<br>-0.08<br>-0.73	
	PAP_00035	3261	3770	-0.21	Succinyl-CoA synthetase(SCS) ,subunit alpha
	PAP_05875	856	1344	0.67	Succinyl-CoA synthetase(SCS) , subunit beta
	PAP_04320<br>-<br>04330	1908<br>9725<br>6827	2338<br>12723<br>10624	-0.29<br>-0.39<br>-0.64	Pyruvate:ferredoxin oxidoreductase（POR）
	PAP_04305<br>-04315	1835<br>10851<br>11573	2656<br>16844<br>21640	-0.53<br>-0.63<br>-0.90	Ketoisovalerate oxidoreductase(VOR)
	PAP_<br>05275	7806	11122	-0.51	Indolepyruvate oxidoreductase,alpha chain (IOR)
	PAP_<br>05280	1704	3463	-1.02	Indolepyruvate oxidoreductase, beta chain (IOR)
	PAP_<br>03610	8326	1974	2.08	Acetyl-CoA synthetase (ADP-forming) Ⅰ ,alpha chain(ACS)
	PAP_<br>05270	3547	4441	-0.32	Acetyl-CoA synthetase (ADP-forming) Ⅱ807,alpha chain(ACS)
	PAP_<br>05870	2338	3209	-0.46	Acetyl-CoA synthetase (ADP-forming) ,alpha chain(ACS)
	PAP_<br>06970	5283	5641	-0.09	Acetyl-CoA synthetase (ADP-forming),beta chain(ACS)
	PAP_<br>03305<br>PAP_03310	8371<br>5260	5357<br>2616	0.64<br>1.01	2-oxoglutarate oxidoreductase(OFOR)<br>2-oxoacid:ferredoxin oxidoreductase (KorAB)
	PAP_<br>09030	582	1159	-0.99	Tungsten-containing aldehyde:ferredoxin oxidoreductase(AOR)
	PAP_<br>08640	418	886	-1.08	Tungsten-containing formaldehyde:ferredoxin oxidoreductase (FOR)
	PAP_03980	699	966	-0.47	Fe-dependent alcohol dehydrogenase (ADH)
	PAP_00835	1634	988	0.73	Phosphoenolpyruvate (PEP) carboxylase (PC)
	PAP_02550	1738	1597	0.12	PEP carboxykinase (PCK)
	PAP_03770	3288	4048	-0.30	Pyruvate carboxylase (PVC)
	PAP_<br>09695	172018	18247	-0.09	Glutamate dehydrogenase（GDH）
	PAP_06440	433	725	-0.71	Pyruvate formate lyase, alpha chain
	PAP_06540	804	1538	-0.94	Pyruvate formate lyase, beta chain
	PAP_03610	2612	2735	-0.07	Alanine aminotransferase (Ala AT)
Protease	PAP_01420	764	884	-0.21	Zn-dependent protease(membrane-bound)
	PAP_01675	1598	517	1.63	Peptidase S8 (extracellular)
	PAP_<br>02280	232	529	-1.19	Transglutaminase-like enzyme, putative cysteine protease,YebA [Posttranslational modification, protein turnover, chaperones] (membrane-bound)

	PAP_03090	374	509	-0.44	Metalloprotease(intracellular)
	PAP_04200	102	372	-1.87	Metalloprotease(membrane-bound)
	PAP_04645 - PAP_04650	230<br>399	471<br>788	-1.03<br>-0.98	Serine protease (ClpP class) [Posttranslational modification, protein turnover, chaperones] (membrane-bound)
	PAP_05230	99	340	-1.78	Zinc-dependent protease (intracellular)
	PAP_05570	77	428	-2.47	Protease (intracellular)
	PAP_09635	607	308	0.98	Peptidase (intracellular)
	PAP_09730	2802	2292	0.29	Protease (membrane-bound)
Peptidase	PAP_00355	2598	2211	0.23	Aminopeptidase (membrane-bound)
	PAP_01300	1234	1783	-0.53	Peptidase (extracellular)
	PAP_01620	749	887	-0.24	Dipeptidase (membrane-bound)
	PAP_02025	279	441	-0.66	Aminopeptidase YpdF (MP-, MA-, MS-, AP-, NP- specific)
	PAP_02935	3707	4371	-0.24	Aminopeptidase YpdF (MP-, MA-, MS-, AP-, NP- specific)
	PAP_02975	992	551	0.85	Peptidase (membrane-bound)
	PAP_03690	2436	3734	-0.62	Peptidase M50, zinc metallopeptidase (membrane-bound)
	PAP_04570	1165	1288	-0.14	Archaeal signal peptidase (membrane-bound)
	PAP_05440	4477	3383	0.40	Deblocking aminopeptidase (EC 3.4.11.-)
	PAP_06815	827	1105	-0.42	Prolyl endopeptidase (EC 3.4.21.26)
	PAP_07055	621	605	0.04	Pyrrolidone-carboxylate peptidase (EC 3.4.19.3)
	PAP_07075	1439	2572	-0.84	Thermostable carboxypeptidase 1 (EC 3.4.17.19)
	PAP_07630	1878	1682	0.16	Methionine aminopeptidase (EC 3.4.11.18)
	PAP_07645	1086	1071	0.02	Isoaspartyl aminopeptidase (EC 3.4.19.5)
	PAP_08040	873	972	-0.15	Deblocking aminopeptidase (EC 3.4.11.-)
	PAP_08100	413	679	-0.72	D-aminopeptidase
Amino-transferase	PAP_03885	204	310	-0.60	Histidinol-phosphate aminotransferase (EC 2.6.1.9)
	PAP_04390	17049	20334	-0.25	4-aminobutyrate aminotransferase
	PAP_04820	3749	8662	-1.21	Aspartate aminotransferase (AspB-4) (EC 2.6.1.1)
	PAP_04910	117	421	-1.85	Pyridoxal-phosphate dependent aminotransferase
	PAP_05920	7429	10788	-0.54	Aspartate aminotransferase (EC 2.6.1.1)
	PAP_06205	1912	1225	0.64	Aspartate aminotransferase (EC 2.6.1.1)
	PAP_06865	1322	1127	0.23	Glucosamine--fructose-6-phosphate aminotransferase [isomerizing] (EC 2.6.1.16)
	PAP_08180	2612	2735	-0.07	Alanine aminotransferase
	PAP_09530	9579	7515	0.35	Serine-glyoxylate aminotransferase (EC 2.6.1.45)
	PAP_09565	209	445	-1.09	Glutamate aminotransferase

	Transporter Amino acid degradation	PAP_ 00150 - 00170	532	653	-0.30	Dipeptide transport system (DppABCDF, APT)
			251	388	-0.63	
			153	354	-1.21	
			151	338	-1.16	
			3646	2687	0.44	
		PAP_03900 - 03925	117	295	-1.33	Branched-chain amino acid ABC transporter(LivKGFHM, AAT)
			26	137	-2.40	
			39	203	-2.38	
			95	279	-1.55	
			125	366	-1.55	
			146	295	-1.01	
		PAP_03785	989	2629	-1.41	Proton/glutamate symport protein(GltT)
		PAP_05545	94	413	-2.14	Sodium/proline symporter, SSF family(PutP)
		PAP_06890	225	1259	-2.48	Proline permease
		PAP_06940	1042	2141	-1.04	Aromatic amino acid permease(Cat-1)
		PAP_09570	160	438	-1.45	Alanine glycine permease(AGCS)
		PAP_06335 - 06355	198	729	-1.88	Dipeptide transport system (DppABCDF, APT)
			48	244	-2.35	
			57	232	-2.03	
			91	331	-1.86	
			193	528	-1.45	
		PAP_10185 - 10210	200	240	-0.26	Oligopeptide transport system, OPT family
			714	958	-0.42	
			640	796	-0.31	
			943	1111	-0.24	
			485	686	-0.50	
			4623	4306	0.10	
		PAP_04080 - PAP_04085	6703	10018	-0.58	Oligopeptide transporter, OPT family
			5031	5267	-0.07	
		PAP_09695	182479	172018	-0.09	Glutamate dehydrogenase
Metabolism of carbohydrates	Starch degradation	PAP_00275	389	533	-0.45	α-amylase（extracellular）,GH13 family
		PAP_01075	1124	921	0.29	Cyclodextrin glucosyltransferase（extracellular）, GH13 family
		PAP_09095	1159	907	0.35	α-amylase（intracellular）,GH13 family
		PAP_09225	2120	1530	0.47	4- α - glucanotransferase（intracellular）， GH57 family
	Maltose and trehalose degradation	PAP_04140	72	368	-2.35	α-galactosidase
		PAP_04145	96	312	-1.70	β- galactosidase
		PAP_05120	7988	11334	-0.50	Trehalose synthase
		PAP_08185	460	918	-1.00	Glycosidase
		PAP_08190	654	1311	-1.00	Glycogen-debranching enzyme
		PAP_05485	9212	13542	-0.56	Amylopullulanase
	ABC-type transport systems	PAP_04995 - 05020	345	595	-0.79	Mal-I, malEFGK，transport maltotriose and longer oligomers
			5972	5078	0.23	
			713	738	-0.05	
			1307	1206	0.12	
			5086	5164	-0.02	

			890	1076	-0.27	
		PAP_05100 - 05110	127<br>34<br>371	419<br>128<br>1019	-1.72<br>-1.91<br>-1.46	Mal-II, transport maltooligosaccharides;
		PAP_05125 - 05135	3597<br>3137<br>28993	4390<br>2360<br>18931	-0.29<br>0.41<br>0.61	Mal-III, recognizes and transports maltose and trehalose;
		PAP_05160 - 05175	182<br>39<br>58<br>82	672<br>235<br>241<br>403	-1.88<br>-2.59<br>-2.05<br>-2.30	Mal-IV, malEFGK，transport maltotriose and longer oligomers
		PAP_05160 - 05175	709<br>64<br>75<br>99	772<br>210<br>202<br>264	-0.12<br>-1.71<br>-1.43<br>-1.42	Sugar ABC-type transport system could transport sugar, eg. N-Acetyl-D-glucosamine and maltose.
		PAP_06890	225	1259	-2.48	Proline permease
		PAP_06940	1042	2141	-1.04	Aromatic amino acid permease
		PAP_02220	15408	6805	1.18	C4-dicarboxylate ABC transporter substrate-binding protein;TRAP transporter solute receptor, TAXI family
		PAP_10245	7903	4042	0.96	Anion permease; low-affinity inorganic phosphate transporter
	Glycerol degradation	PAP_03945	63	280	-2.15	Glycerol transport protein
		PAP_02595 - 02605	213<br>90<br>214	314<br>375<br>472	-0.56<br>-2.06<br>-1.14	Glycerol kinase<br>Glycerol-3-phosphate dehydrogenase<br>Glycerophosphoryl diester phosphodiesteras（glpQKAD）
		PAP_08525	152	330	-1.12	Glycerol kinase(glpK)
		PAP_08540	81	279	-1.78	Glycerol-3-phosphate dehydrogenase(glpA)
Nucleic acid biosynthesis	Purine Biosynthesis	PAP_00540 - 00605	275<br>89<br>24<br>132<br>202<br>50<br>59<br>26<br>148<br>60<br>178<br>26<br>201<br>69	678<br>179<br>58<br>308<br>382<br>85<br>159<br>87<br>432<br>199<br>153<br>27<br>328<br>217	-1.30<br>-1.01<br>-1.27<br>-1.22<br>-0.92<br>-0.77<br>-1.43<br>-1.74<br>-1.55<br>1.73<br>0.22<br>-0.05<br>-0.71<br>-1.65	Phosphoribosylformylglycinamidine synthase,<br>Pur LSPDETMCFOPC
	GMP synthase	PAP_00690	314	470	-0.58	GMP synthase
		PAP_00695	123	247	-1.01	GMP synthase
Coenzyme biosynthesis	Riboflavin biosynthesis	PAP_00630 - 00600	79<br>44<br>98<br>51	433<br>221<br>516<br>143	-2.45<br>-2.33<br>-2.40<br>-1.49	Riboflavin synthase, RibDEBAH
		PAP_05950	123	257	-1.06	Riboflavin synthase, RibL

	Thiamin biosynthesis	PAP_03460	176	255	-0.53	Thiamin-monophosphate kinase(ThiL)
		PAP_03985	59	125	-1.08	Hydroxyethylthiazole kinase(ThiM)
		PAP_03990	37	115	-1.64	Thiamin phosphate synthase(ThiE)
		PAP_03995	74	221	-1.58	Bifunctional hydroxymethylpyrimidine kinase/phosphohydroxymethylpyrimidine kinase(ThiD)
		PAP_00665	150	255	-0.77	Thiamin (pyrimidine moety) biosynthesis protein(ThiC) (Cys residues near extreme C-terminus)
		PAP_05095 - 05100	34<br>371	128<br>1019	-1.91<br>-1.46	Thiamin ABC transport system
	Cobalamin biosynthesis	PAP_06425	3373	253	3.74	Cobalamin biosynthesis protein CobQ; 4Fe-4S ferredoxin type; iron-sulfur cluster binding domain
		PAP_06430	2931	276	3.41	Cobalamin biosynthesis protein CobQ; 4Fe-4S ferredoxin type; iron-sulfur cluster binding domain
Energe metabolism	Hydrogenase	PAP_01095 - 01160	3434<br>1479<br>2178<br>1266<br>2181<br>2439<br>2918<br>9511<br>1531<br>1988<br>3249<br>9654<br>6292<br>3722	1792<br>951<br>1600<br>777<br>1254<br>1518<br>1756<br>6318<br>700<br>1233<br>2501<br>7778<br>6634<br>3997	0.94<br>0.64<br>0.44<br>0.70<br>0.80<br>0.68<br>0.73<br>0.59<br>1.13<br>0.69<br>0.38<br>0.31<br>-0.08<br>-0.10	Membrane-bound proton-reducing H2-evolving hydrogenase complexes (MBH1)
		PAP_01165 - 01230	387<br>195<br>182<br>97<br>132<br>128<br>163<br>608<br>64<br>106<br>206<br>364<br>295<br>206	388<br>202<br>208<br>111<br>169<br>206<br>207<br>1038<br>125<br>157<br>311<br>627<br>647<br>319	0.00<br>-0.05<br>-0.19<br>-0.19<br>-0.36<br>-0.69<br>-0.34<br>-0.77<br>-0.97<br>-0.57<br>-0.59<br>-0.78<br>-1.13<br>-0.63	Membrane-bound proton-reducing H2-evolving hydrogenase complexes (MBH2)
		PAP_02355 - 02415	2448<br>938<br>1214<br>915<br>2716;<br>982;<br>4993;	1364<br>408<br>398<br>216<br>741;<br>234;<br>807;	0.84<br>1.20<br>1.61<br>2.08<br>1.87<br>2.07<br>2.63	Membrane-bound NADP-reducing hydrogenase(MBX)

			7346;<br>2988;<br>1490;<br>1958;<br>4088;<br>2083	994;<br>502;<br>170;<br>322;<br>599;<br>440	2.89<br>2.57<br>3.13<br>2.60<br>2.77<br>2.24	
		PAP_01490 - 01505	160;<br>85;<br>129;<br>118	513;<br>202;<br>331;<br>315	-1.68<br>-1.25<br>-1.36<br>-1.42	Cytoplasmic (Ni-Fe) hydrogenases (SHI,sulfI)
		PAP_03240 - 03255	8456;<br>5139;<br>5285;<br>8943	5100;<br>4213;<br>5334;<br>13605	0.73<br>0.29<br>-0.01<br>-0.61	Cytoplasmic (Ni-Fe) hydrogenases (SHII,sulfII)
	Hydrogenase maturation	PAP_ 01595	32502	3010	3.43	Ni-Fe hydrogenase metallocenter assembly protein, HypF;
		PAP_ 04030	2840	883	1.69	hydrogenase expression/formation protein, HypE
		PAP_ 06285	7855	4813	0.71	hydrogenase isoenzymes formation protein, HypD
		PAP_06290	1187	388	1.61	Ni-Fe hydrogenase maturation protein,HypC
		PAP_ 08995	1873	478	1.97	Ni-Fe hydrogenase nickel incorporation protein ,HypA
		PAP_03260	585	669	-0.19	Hydrogenase maturation protease,HybD
		PAP_09005	1086	240	2.18	Hydrogenase maturation protease,HycI
	Oxi-reductase	PAP_ 08740	65	216	-1.73	NADH-cytochrome b5, Heme/Steroid binding domain
		PAP_01510 - PAP_ 01525	55<br>399<br>392<br>89	61<br>589<br>1002<br>247	-0.15<br>-0.56<br>-1.35<br>-1.47	NADH-quinone oxidoreductase
		PAP_06370	208	341	-0.71	NAD(P)H-flavin oxidoreductase
		PAP_05150 - PAP_05155	79<br>84	270<br>289	-1.77<br>-1.78	Oxidoreductase，NAD(P)-binding domain
	ATP synthase	PAP_09395 - 09435	508<br>5548<br>906<br>1100<br>1877<br>347<br>4096<br>3766<br>1004	509<br>6880<br>1243<br>1234<br>1862<br>249<br>3136<br>3080<br>847	0.00<br>-0.31<br>-0.46<br>-0.17<br>0.01<br>0.48<br>0.39<br>0.29<br>0.25	V-type ATP synthase subunit(ATPVABCDEFGIK)
Inorganic ion transport and metabolism		PAP_05880 - 05910	176<br>64<br>309<br>191<br>1057<br>40<br>4394	478<br>400<br>928<br>411<br>2311<br>102<br>10118	-1.44<br>-2.64<br>-1.59<br>-1.11<br>-1.12<br>-1.35<br>-1.20	Facilitator transporter

		PAP_06115 - PAP_ 06120	51<br>98	168<br>271	-1.72<br>-1.47	ABC-type multidrug transport system，permease and ATPase
		PAP_06800	514	928	-0.99	Sodium-driven multidrug efflux pump protein
		PAP_07815	43	411	-2.62	Low-affinity inorganic phosphate transporter
		PAP_08130 - PAP_ 08135	213<br>294	430<br>682	-1.01<br>-1.21	ABC-type multidrug transport system，permease and ATPase
		PAP_01680 - 01695	528<br>269<br>307<br>581	152<br>107<br>179<br>474	1.80<br>1.33<br>0.78<br>0.29	Nickel/Cobalt ABC transporter (cbiMNQO)
		PAP_08930	1268	714	0.83	EfeU-like ferrous iron transport permease
Cell motility and secretion		PAP_04440 - 04485	131<br>733<br>76<br>50<br>14<br>55<br>285<br>149<br>369	887<br>3983<br>211<br>208<br>33<br>112<br>543<br>377<br>783	-2.76<br>-2.44<br>-1.47<br>-2.06<br>-1.24<br>-1.03<br>-0.93<br>-1.34<br>-1.09	Flagella protein (flaABCDGHIJ)
		PAP_ 07020	498	746	-0.58	UDP-N-acetylglucosamine 2-epimerase(WecB)
		PAP_ 07025	1041	1014	0.04	UDP-N-acetyl-D-mannosaminuronic acid dehydrogenase(WecC)
Sulfur metabolism	Sulfur reduction	PAP_ 01275	4099	1723	1.25	Coenzyme A-dependent NAD(P)H elemental sulfur oxidoreductase(NSR)
		PAP_ 03325	2442	906	1.43	Pyridine nucleotide-disulfide oxidoreductase（NPSOR）
		PAP_00200 - PAP_ 00205	9127<br>8657	2745<br>1899	1.73<br>2.19	Ferredoxin-NADP$^+$ reductase（SuDHI）
		PAP_ 01255 PAP_01260	125<br>250	219<br>522	-0.81<br>-1.06	Sulfate-transporting ATPase;sulfate transporter
		PAP_07225	7280	8810	-0.28	Glutarredoxin(pdo)
		PAP_08980	641	611	0.07	Glutarredoxin
	Sulfate reduction	PAP_09885	1132	572	0.98	Sulfate adenylyltransferase(Sat)
		PAP_09900	282	269	0.07	Adenylylsulfate kinase(cysC)
		PAP_04545	1413	1282	0.14	PAPS reductase (cysH)
	Cystein biosynthesis	PAP_05900	1057	2311	-1.13	Cysteine synthase(cysk,OASS)
		PAP_08745	87	631	-2.86	Cystathionine beta-synthase
		PAP_08750	144	631	-2.13	Cystathionine gamma-lyase
		PAP_06715	2586	2495	0.05	Cysteinyl-tRNA synthetase
		PAP_01245	2080	2938	-0.15	Cysteine desulfurase(SufS)

	Fe-S cluster biogenesis	PAP_02345	308	586	-0.93	Fe-S cluster assembly ABC transporter (SufC)
		PAP_02350	866	1274	-0.56	Fe-S cluster containing protein(SufB)
		PAP_08965	6017	6692	-0.50	Cysteine desulfurase (iscS)
		PAP_ 08975	939	1100	-0.23	NifU-like Fe-S cluster assembly scaffold protein (IscU)
		PAP_ 09000	1086	240	2.18	Cytosolic Fe-S cluster carrier, Mrp/NBP35 ATP-binding protein
	Fe–S cluster-containing proteins	PAP_06515	1980	101	4.29	Iron-molybdenum cofactor-binding protein
		PAP_06520	15920	212	6.23	Fe-Mo cluster-binding protein(SipA,PF2025)
		PAP_00320 - PAP_ 00330	228<br>36622<br>3605<br>1374	110<br>11960<br>1311<br>630	1.05<br>1.61<br>1.46<br>1.12	Radical SAM protein, iron-sulphur containing
Iron uptake	Iron transporter	PAP_ 03965	61	145	-1.25	ABC-type iron (III) transporter ATPase(ABC.FEV.A)
		PAP_ 03970	73	162	-1.15	ABC-type iron (III) transporter permease(ABC.FEV.P)
		PAP_ 03975	542	667	-0.30	ABC-type iron (III) transporter Periplasmic binding protein(ABC.FEV.S)
		PAP_ 06035	3912	515	2.93	ABC-type iron(III)-siderophore transporter permease(ABC.FEV.P)
		PAP_ 06040	3065	430	2.83	ABC-type iron(III)-siderophore transporter ATPase(ABC.FEV.A)
		PAP_ 06225	5076	2237	1.18	Iron III ABC transporter Periplasmic binding protein (ABC.FEV.S)
		PAP_ 08650	378	261	0.53	ABC-type iron(III)-siderophore transporter ATPase (ABC.FEV.A)
		PAP_ 08655	454	237	0.94	ABC-type iron(III)-siderophore transporter permease (ABC.FEV.P)
		PAP_ 08660	1795	427	2.07	ABC-type iron (III) transporter Periplasmic binding protein (ABC.FEV.S)
		PAP_ 06060	5734	1852	1.63	Ferrous iron transporter (feoB)
		PAP_ 06065	283	138	1.04	Ferrous iron transporter (feoA)
		PAP_ 08930	1268	714	0.83	EfeU-like ferrous iron transport permease
		PAP_ 04295	90	316	-1.81	Low-affinity inorganic phosphate transporter (pltA)
		PAP_ 07815	43	264	-2.61	Low-affinity inorganic phosphate transporter
		PAP_ 10245	7903	4042	0.96	Low-affinity inorganic phosphate transporter
		PAP_ 03235	11891	27659	-1.22	Formate transporter
	Storge	PAP_ 08895	715	891	-0.32	Iron storge protein, ferritin-like protein

	PAP_ 06370	208	341	-0.71	NADPH–flavin oxidoreductase
Regulators	PAP_ 02730	1095	541	1.02	Transcription termination protein NusA ,NusA-like transcription elongation factor,NusA superfamily
	PAP_ 09615	1638	786	1.06	XRE family transcriptional regulator，containing helix-turn-helix domain，COG1709 superfamily
	PAP_ 09880	199	90	1.14	CopG transcriptional regulator,RHH_1 superfamily
	PAP_ 05280	526	604	-0.20	TBP, TATA-box-binding protein(TON_1309)
	PAP_ 08070	742	2627	-1.82	Transcription regulator TrmB (TON_0332)
	PAP_ 04990	1439	944	-0.61	Transcription regulator TrmB(TON_1797)
	PAP_ 06755	287	325	-0.18	Transcription regulator AsnC-type (TON_0662)
	PAP_ 05565	77	428	-2.47	Transcription regulator AsnC-type (TON_1284)
	PAP_ 00110	340	276	0.30	Transcription regulator AsnC-type (TON_1510)
	PAP_ 08835	56	250	-2.15	ArsR-type transcription regulator, HTH motif
	PAP_ 06395	682	1689	-1.30	ArsR-type transcriptional regulator
	PAP_ 03045	48	114	-1.25	ArsR-type DNA binding domain (SurR, PF2051)
	PAP_ 07220	1521	1857	-0.28	ArsR-type DNA binding domain(PF0095)
Oxygen Detoxificatio n	PAP_ 03325	2442	906	1.43	NAD(P)H:rubredoxin oxidoreductase (NROR)
	PAP_ 03330	2211	2988	-0.43	Rubrerythrin
	PAP_ 03335	1148	1696	-0.56	Superoxide reductase（SOR）
Stress responce	PAP_ 04835	1168	928	0.33	Heat shock protein
	PAP_ 07685	1688	871	0.95	Heat shock protein
	PAP_ 08375	929	976	-0.07	Heat shock protein
	PAP_ 06790	216	663	-1.62	Heat shock regulator
	PAP_ 06210	41	133	-1.70	DNA repair and recombination protein RadB
	PAP_ 06965	1484	4458	-1.59	Restriction endonuclease
	PAP_07940 - 07950	27<br>117<br>53	82<br>347<br>104	-1.60<br>-1.57<br>-0.97	CRISPR-associated protein(cas1.cas2,cas4a)

	PAP_03320	2211	2988	-0.43	Rubrerythrin
	PAP_03360	709	895	-0.34	Rubrerythrin
	PAP_03370	57	123	-1.11	Rubrerythrin
	PAP_03375	281	224	0.33	Rubrerythrin
	PAP_03385	96	207	-1.11	Rubrerythrin
	PAP_03425	163	350	-1.10	Rubrerythrin
	PAP_03330	522	465	0.17	Rubredoxin
	PAP_03335	1148	1696	-0.56	Desulfoferrodoxin, ferrous iron-binding region
Others	PAP_06140 - 06190	9<br>24<br>112<br>64<br>26<br>110<br>67<br>21<br>35<br>67	19<br>80<br>387<br>139<br>67<br>267<br>286<br>144<br>166<br>237	-1.08<br>-1.74<br>-1.79<br>-1.12<br>-1.37<br>-1.28<br>-2.09<br>-2.78<br>-2.25<br>-1.82	Hypothetical protein
	PAP_07315-07350	45<br>66<br>11<br>48<br>85<br>95<br>35<br>43	181<br>229<br>21<br>176<br>164<br>182<br>68<br>104	-2.01<br>-1.79<br>-0.93<br>-1.87<br>-0.95<br>-0.94<br>-0.96<br>-1.27	Methyltransferase(UbiE)
	PAP_06505	1141	553	1.04	Methyltransferase(UbiE)
	PAP_00080	2777	1209	1.20	(S)-2,3-di-O-geranylgeranylglyceryl phosphate synthase (UbiA)
	PAP_04045	1170	136	3.10	Hypothetical protein
	PAP_09525	9740	2169	2.17	Ribonucleoside-diphosphate reductase
	PAP_09010	1420	381	1.90	Electron transporter; cytochrome complex assembly

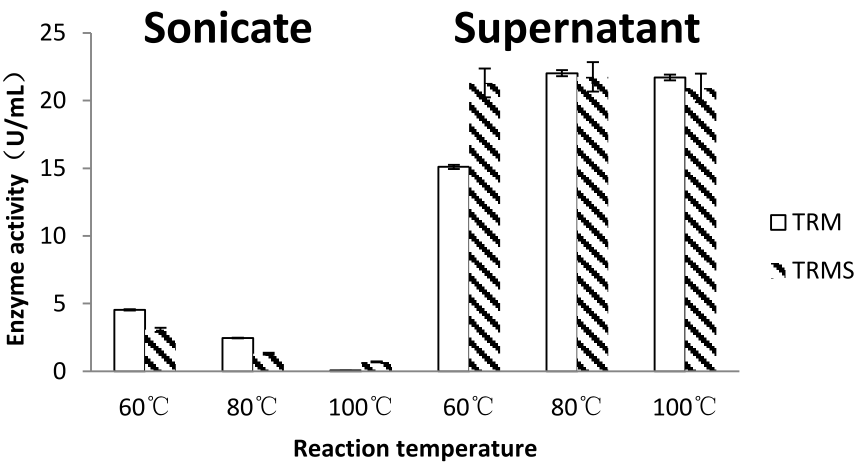

Figure S1. Protease activity at different temperature from cell sonicate or supernatant from strain DY20341 cultured in different medium TRM and TRMS
